# FAM117A Is a New Prognostic Marker of Lung Adenocarcinoma and Predicts Sensitivity to PD0332991

**DOI:** 10.1155/2022/3945446

**Published:** 2022-03-03

**Authors:** Chao Wu, Jiajin Zhang, Kuan Wang, Mengjiao Fan, Yi Hu

**Affiliations:** ^1^Department of Oncology, Chinese PLA General Hospital, Beijing 100853, China; ^2^Department of Systems Surgery, Chinese PLA General Hospital, Beijing 100853, China; ^3^Department of Respiratory Medicine, Fangshan Hospital, Beijing University of Chinese Medicine, Beijing 102400, China

## Abstract

Lung cancer is the second most common cancer and the leading cause for cancer mortality worldwide. Accelerated cell cycle progression is a well-characterized hallmark for cancer. The present study aims to identify biomarkers for clinical outcomes of lung cancer patients and their sensitivity to CDK inhibitors. To this end, bioinformatics analysis of transcriptome datasets from the Cancer Genome Atlas (TCGA) was first performed to identify survival-related genes; cell proliferation assay, colony formation assay, flow cell cytometry, western blot, EDU labelling, and xenograft models were then used to confirm the potential roles of the identified factors. Our results identified the decreased FAM117A expression as the most significant survival related factor for poor outcome. The cell cycle transition from G1 to S phase was suppressed upon FAM117A overexpression and was promoted upon FAM117A knockdown. Accordingly, the tumor cell growth induced by FAM117A depletion was completely blocked by treatment with PD0332991, which has been approved for cancer therapy. In summary, our work identified FAM117A as a new prognostic marker for poor outcomes of lung cancer patients, predicting sensitivity to PD0332991 treatment.

## 1. Introduction

Lung cancer is the second most diagnosed cancer and the leading cause for cancer mortality worldwide, causing more than 1.8 million deaths in 2020 [1]. Nonsmall lung cancer (NSCLC) represents the major histological subtype of lung cancer, accounting for about 85% of all the diagnosed cases [2]. NSCLC was further classified into lung adenocarcinoma (LUAD), lung squamous cell carcinoma (LUSC), and large cell carcinoma, of which LUAD is the most common subtype with highly heterogenicity and malignancy [3]. Furthermore, mutations in driver genes were frequently observed in LUAD patients, such as epidermal growth factor receptor (EGFR), Kirsten ras oncogene (KRAS) [4], erb-b2 receptor tyrosine kinase 2 (ERBB2), tumor protein p53 (TP53), anaplastic lymphoma kinas (ALK), and serine/threonine kinase 11 (STK11), leading to sustained growth promoting signals as well as resistance to cell death [2, 5]. Tobacco smoking has been well defined as the most common etiology of lung cancer among all subtypes, causing more than 80% of all cancer-related deaths [2]. In addition, inherent genetics and exposure to carcinogens have also been well characterized as risk factors for lung cancer worldwide [6].

Considerable progress has been made in clinical practice during the past decade due to improved understanding of pathological genetic mutations, the development of targeted drugs, as well as characterized markers for immune checkpoint inhibitors [7–9]. However, cytotoxic chemotherapy remains the major choice for most of the cases, and the five-year survival for advanced stage patients was less than 15% [10, 11]. Therefore, improvements in lung cancer diagnostics and new treatments are urgently needed.

Inhibitors of the cell cycle dependent kinases have been successfully used to treat breast cancer, and representative inhibitors have been approved for treatment of hormone-receptor-positive breast cancer [12, 13]. PD0332991 is the first CDK inhibitor developed by the Pfizer [12]. Mechanically, CDK4/6 inhibition leads to cell cycle arrest at G0/G1 phase followed by cell death due to long lasting block [14–17]. However, for the other types of cancer, no markers were available to predict their sensitivity to those inhibitors.

The family with sequence similarity 117 member A (FAM117A) is a C/EBP-induced protein with unknown functions and was reported as a DYRK1A interacting protein promoting the recruitment of 53BP1 to DNA damage sites [18–20]. In the present study, for the first time, we identified FAM11A as a tumor suppressor and prognostic marker in LUAD patients, leading to cell cycle arrest at G0/G1 phase and predicting sensitivity to cyclin-dependent kinase inhibition.

## 2. Materials and Methods

### 2.1. Cells and Reagents

The A549 (1101HUM-PUMC000002) and NCI-H1975 (1101HUM-PUMC000252) cells were from the National Infrastructure of Cell Line Resource (NICR). Antibodies for cyclin D (ab207604), phospho-CDK4 (ab277788), CDK4 (ab108357), p21 (ab109520), p27 (ab32034), FAM117A (ab172714), and beta-actin (ab6276) were purchased from Abcam. The antibody for Flag (F1804) was from Sigma. The EDU (ab146186) was from Abcam. The medium and the fetal bovine serum (FBS, 10100147) for cell culture were from ThermoFisher.

### 2.2. Cell Proliferation Assay

Cells were seeded into a 96-well plate at a concentration of about 3000 cells per well. To determine the activity of the cells, 20 *μ*l cell counting kit 8 (CCK-8) reagent was added into the complete culture medium and incubated at 37 degrees for about 1 hour, and then was quantitated by collecting absorbance at OD450 nm. All the treatments were performed with 3 repeats. Data was analyzed by GraphPad Prism 6.01. All the experiments were performed in triplicate.

### 2.3. Cell Cycle Profile

To determine the cell cycle profiles, cells were first collected by trypsinization. The complete medium with FBS was removed by wash with PBS twice, followed by fixation with ice-cold 70% ethanol. Subsequently, fixed cells were isolated by centrifugation (1000 *g*, 5 minutes) and were then washed by 1X PBS (1000 *g*, 2 minutes). The cells were then stained with propidium containing RNase at 37 degrees for 30 minutes, and were then analyzed by flow cell cytometry (BD, C6). All the experiments were performed in triplicate. Cell cycle profiles were analyzed by FlowJo *X*.

### 2.4. Colon Formation

About 1000 cells were seeded into a plate (diameter: 6 cm) and were cultured for about 2–3 weeks. The complete medium was changed every 3 days. At the end of the culture, cells were gently washed with PBS, then fixed with methanol, followed by staining with crystal violet blue for about 30 minutes. Cells were imaged and quantitated by counting eye visible colonies. All the experiments were performed in triplicate.

### 2.5. Western Blot

Total cell lysate was prepared by lysing cells with NP-40 containing buffer, followed by centrifugation at 13000 rpm for 10 minutes at 4 degrees. Protein concentration was determined by bicinchoninic acid (BCA, Micro-Helix, EWPQ-0803). About 30 micrograms of total protein were loaded and separated by SDS-PAGE. The separated proteins were transferred onto PVDF. The blot strips were first blocked by 5% nonfat milk in 0.05% PBST at room temperature for 1 hour, followed by incubation with indicated antibodies at 4 degrees overnight. The blots were then washed with 0.05% PBST to remove the unbound primary antibodies and were incubated with secondary antibodies at room temperature for about 1 hour. Repeated washing with 0.05% PBST was performed to remove the unbound secondary antibodies, and the blots were then treated with ECL solution (Micro-Helix, E1248), and the signals were imaged by Tanon 5200-multi.

### 2.6. Lentivirus Package

HEK293 cells were cultured with DMEM supplemented with 10% FBS and 1% penicillin-streptomycin liquid (Gibco). For the lentivirus package, HEK293 cells were transfected (SuperFectin, EWFC004) with 3 lentiviral vectors (psPAX2 and PMD2.G) together. The supernatant was collected by centrifuge 48 hours posttransfection. The lentiviruses were concentrated by the PEG-8000 based method. The vector expressing the FAM117A coding sequence (XM_038611631.1) was cloned into pCDHL by recombination. The lentivirus vectors expressing short hairpin oligos targeting FAM117A were constructed by annealing the synthesized paired oligos and ligated them into the PLKO vector. The sequences for shRNA oligos were as follows: shFAM117A-1#: CCG GGC TGC TGA GGA TCC TTG ATA TCT CGA GAT ATC AAG GAT CCT CAG CAG CTT TTT G; shFAM117A-2#: CCG GAT CAT AGC TAC ATC TTC AAA CCT CGA GGT TTG AAG ATG TAG CTA TGA TTT TTT G.

### 2.7. Stable Cell Lines

Lung cancer cells were transduced with lentiviruses expressing genes coding FAM117A CDS or short hairpin RNA targeting its coding region or 3'-untranslated regions. Cells were treated with 1 *μ*g/ml puromycin for 2 or 3 days, 48 hours posttransduction. The expression of exogenously expressed genes was confirmed by western blot or quantitative real-time polymerase chain reaction.

### 2.8. Quantitative Real-Time PCR

Total RNA was isolated from the lung cancer cells by the TRIzol-chloroform-based method. In brief, adherent lung cancer cells were first washed by PBS and then collected by adding 1 mL of TRIzol. 200 *μ*L of chloroform was thoroughly mixed with the TRIzol lysed cell for 15 seconds followed by centrifugation. Secondly, the aqueous phase was mixed with an equal volume of isopropanol and then stored at -20°C for 30 minutes to precipitate the RNA. The pellet was further isolated by centrifugation and was washed with 75% ethanol diluted with RNase-free water. The concentration of RNA was determined by absorbance at A260. The first stand of cDNA was synthesized by reverse transcriptase using 2 *μ*g of purified total RNA as a template. SYBR Green was used for quantitative real-time RT-PCR, and GAPDH was used as an internal control. The PCR primers for amplification were as follows: FAM117A: sense, 5′- GCT CAG CCT TCT GCC CCG TC-3′; antisense, 5′- GGT GCG CTG CCA TGG CTC G-3′; GAPDH: sense, 5′- GAA GGT GAA GGT CGG AGT C-3′; and antisense, 5′-GAA GAT GGT GAT GGG ATT TC-3. All the experiments were performed in triplicate.

### 2.9. Statistical Analysis

All the statistical analysis was performed by GraphPad Prism (6.01), and the *t*-test and one-way ANOVA were performed to calculate the significance among groups. ^*∗∗*^represents *p*  <  0.01 and ^*∗*^represents *p*  <  0.05.

## 3. Results

### 3.1. FAM117A Is a Prognostic Marker for LUAD Patients and Decreased in Cancer Tissue

**Figure 1:**
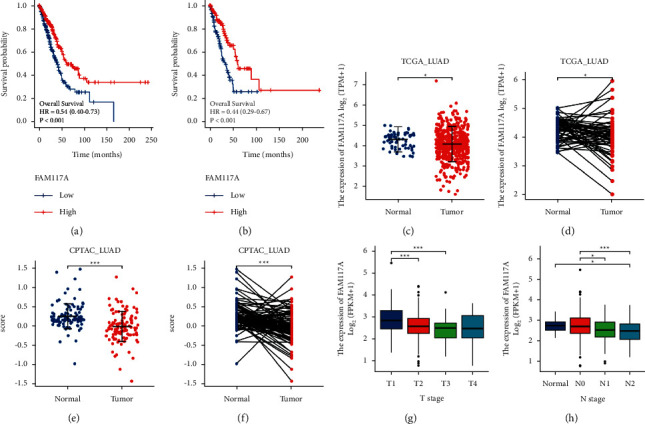
FAM117A expression is decreased in lung cancer patients. (a) Kaplan–Meier overall survival curve comparing the high (50%–100%, *N* = 267) and low (0%–50%, *N* = 268) expression value of FAM117A (determined by the quantile value) for the TCGA LUAD patient cohort. (b) Kaplan–Meier overall survival curve comparing the high (50%–100%, *N* = 134) and low (0%–50%, *N* = 135) expression value of FAM117A (determined by the quantile value) for the TCGA LUAD patient cohort. (c) Dot plot of the FAM117A mRNA expression between lung cancer tissue (*N* = 535) and adjacent normal tissue (*N* = 59). (d) Dot plot of the FAM117A mRNA expression between paired lung cancer tissue (*N* = 59) and adjacent normal tissue (*N* = 59). (e) Dot plot of the FAM117A protein expression between lung cancer tissue (*N* = 111) and adjacent normal tissue (*N* = 102). (f) Dot plot of the FAM117A mRNA expression between paired lung cancer tissue (*N* = 102) and adjacent normal tissue (*N* = 102). (g) Box plot of FAM117A expression among patients of different T stages (T1: *N* = 175; T2: *N* = 289; T3: *N* = 49; T4: *N* = 19). (h) Box plot of FAM117A expression among patients of different N stages (Normal: *N* = 59; N0: *N* = 348; N1: *N* = 95; N2: *N* = 74).

In order to explore molecular mechanisms of lung cancer tumorigenesis, we performed cox regression analysis of a transcriptome dataset retrieved from the TCGA (The Cancer Genome Atlas) program, to identify genes that are associated with the survival of LUAD patients. Interestingly, FAM117A emerged as the most significant prognostic marker for poor outcomes of LUAD survival (Figures 1(a) and 1(b), Supplemental Table 1), with a cox coefficient factor of −0.4743 (*p* = 0.000000023). To determine the potential involvement of FAM117A in lung cancer development, we first analyzed the expression pattern of FAM117A at transcript level in lung cancer tissue and normal tissues using a transcriptome dataset from the TCGA database. Significant decreased FAM117A expression was observed in cancerous tissues versus normal lung tissues, and a similar expression pattern was also observed in paired tissues (Figures 1(c) and 1(d)). Decreased expression of FAM117A in cancerous tissues was also observed in other types of cancers (Supplemental Figure 1). In addition, the decreased expression trend was also observed at the protein level as revealed by the data from the Clinical Proteomic Tumor Analysis Consortium (CPTAC) (Figures 1(e) and 1(f)). Collectively, the expression of FAM117A is significantly decreased in lung cancer tissue. Further data collection was required to determine exactly how FAM117A expression affects lung cancer development. To this end, the Kruskal–Wallis test was performed to explore the correlation between FAM11A expression and clinical features for LUAD patients. As can be seen from Supplemental Table 2, positive statistical correlation was observed with pathological T stage as well as N stage (Figures 1(g) and 1(h)), but no significance was detected with other clinical features including pathology M stage, tumor purity, residual tumor race, and ethnicity.

### 3.2. Downregulated FAM117A Expression Is Associated with Cell Cycle Promoting Pathways

**Figure 2:**
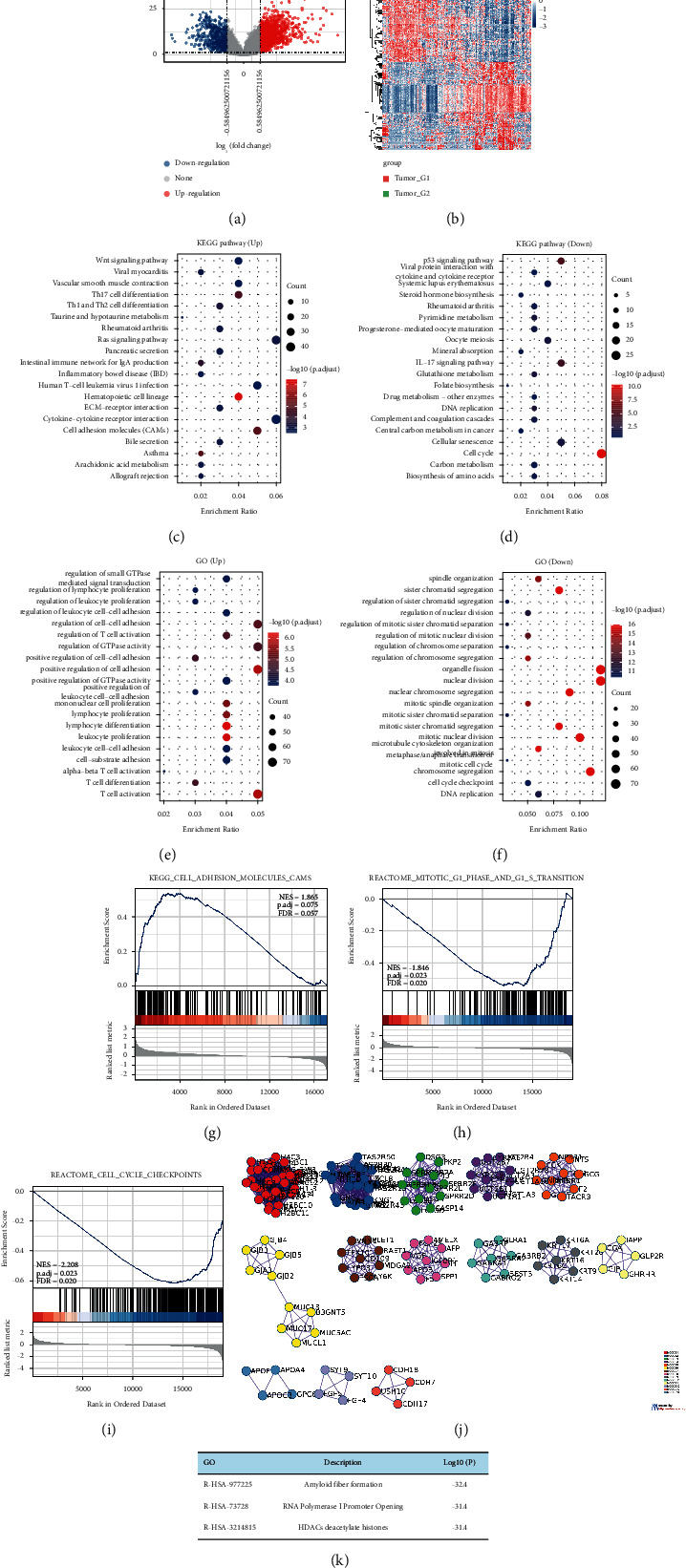
Identification of differentially upregulated expressed genes. (a) Volcano plot of mRNA expression changes between LUAD samples harboring FAM117A high- and low- expression value. The *X*-axis specifies the fold-change (FC) and the *Y*-axis specifies the negative logarithm to the base 10 of the adjusted *p* values. (b) Heatmap of differentially expressed genes between LUAD samples harboring FAM117A high- and low-expression values. (c, d) KEGG Gene Ontology enrichment analysis for upregulated (c) and downregulated (d) genes, respectively. (e, f) KEGG enrichment analysis for upregulated (E) and downregulated (f) genes, respectively. (g, i) Gene set enrichment analysis between FAM117A high- and low-expression lung cancer samples. (j, k) Protein-protein interaction network and MCODE components identified in upregulated genes with low FAM117A expression.

To better understand the molecular mechanism underlying the tumor suppressive roles of the FAM117A transcript in lung cancer development, differentially expressed gene cohorts between patients with high FAM117A (*n* = 257) expression and low FAM117A expression (*n* = 256) were analyzed. As shown in the volcano plots of Figure 2(a), 1657 upregulated genes and 637 downregulated genes were identified in patients with high FAM117A expression. To further confirm the molecular functions of all the dysregulated genes, Kyoto Encyclopedia of Genes and Genomes (KEGG) enrichment was employed to identify associated high-level genome functional information. Interestingly, upregulated genes in patients with high FAM117A expression were mainly enriched in cytokine-cytokine receptor interaction (ko04060), cell adhesion molecules (CAMs) (ko04514), hematopoietic cell lineage (ko04640), and Th17 cell differentiation (ko04659) (Figure 2(c)). On the contrary, the most significant pathways enriched in patients with lower FAM117A expression were cell cycle (ko04110) (Figure 2(d)), suggesting a higher proliferation rate of tumor cells in patients with low FAM117A expression. Consistently, further evidence as demonstrated by the Gene Ontology analysis of the differentially expressed genes, predicted the potential role of FAM117A as a tumor suppressor in lung cancers (Figures 2(e) and 2(f)). In addition, gene set enrichment analysis (GSEA) enriched genes set (KEGG_CELL_ADHESION_MOLECULES_CAMS) positively correlated with FAM117A expression as shown in Figure 2(g), and negatively correlated pathways such as G1/S transition as well we cell cycle checkpoint (Figures 2(h) and 2(i)), suggesting that FAM117A might be a negative regulator for cell cycle progression. Further evidence from the protein-protein interaction network revealed that most of the downregulated genes were related with the RNA polymerase I promoter opening complex (Figures 2(j) and 2(k)). Collectively, the absence of FAM117A expression in lung cancer cells is highly correlated with accelerated cell cycle progress and might be a tumor suppressor.

### 3.3. Expression of FAM117A Is Tightly Related with Lung Cancer Cell Cycle Progression and Growth In Vitro and In Vivo

**Figure 3:**
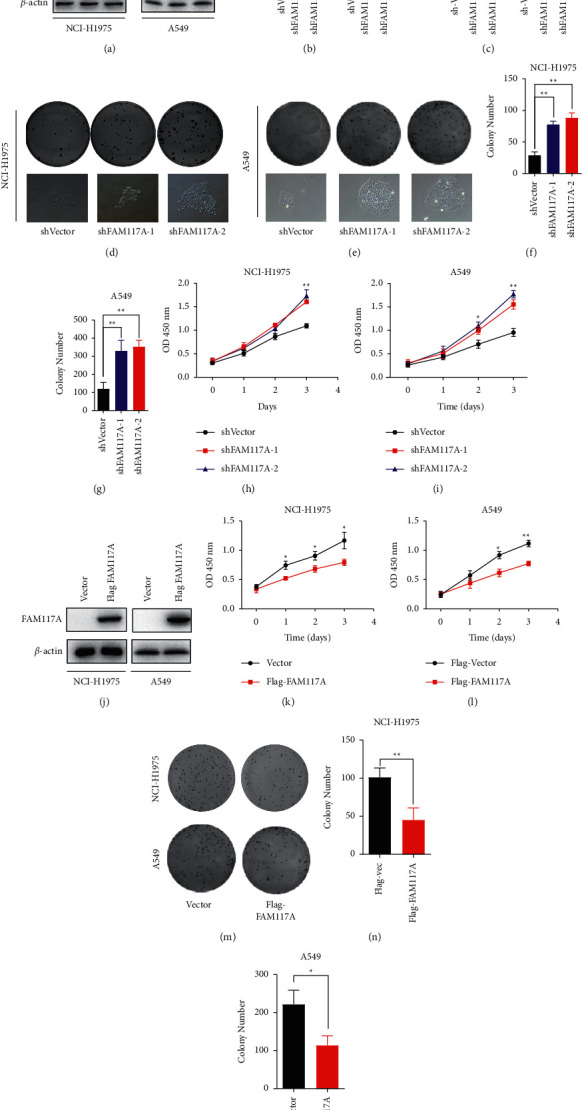
Effects of FAM117A expression in lung cancer cells. (a) Images of western blot results of FAM117A expression in lung cancer cell lines (NCI-H1975 and A549) upon transfection with short hairpin RNA oligos targeting the FAM117A transcript, *β*-actin was used as an internal control. (b) Quantitative analysis of the grey scale of FAM117A expression by western blot. (c) Quantitative real-time RT-PCR of FAM117A expression in lung cancer cells transfected with shRNA oligo targeting its transcript. (d, e) Images of colony formation for lung cancer cells knockdown with FAM117A. (f, g) Quantitative results of the colony formation assay in lung cancer cells. (h, i) Lung cancer cell proliferation rate as confirmed by CCK-8 based assay for 3 days post seeding in 96-well plate. (j) Images of western blot results of FAM117A overexpression in lung cancer cell lines (NCI-H1975 and A549) upon transfection with Flag-FAM117A, *β*-actin was used as an internal control. (k, l) Lung cancer cell proliferation rate as confirmed by CCK-8 based assay for 3 days postseeding in 96-well plate. (m, o) Images of colony formation and quantitative results for lung cancer cells overexpressed with the empty vector of Flag-FAM117A.

To further confirm the tumor suppressor roles of FAM117A in lung cancer cells, we next modulated its expression by lentivirus-based overexpression or RNA interference targeting its coding region. The expression of FAM117A was confirmed at both the protein level (western blot) and transcript level (quantitative real-time PCR) (Figures 3(a)–3(c)). As can be seen from Figures 3(d)–3(g), FAM117A knockdown in lung cancer cell lines (A549 and NCI-H1975) significantly promoted proliferation as demonstrated by increased number and size of colonies, which was further confirmed by the CCK-8 assay (Figures 3(h) and 3(i)). In contrast to what was observed in the FAM117A silenced cells, the growth of lung cancer cells with elevated FAM117A expression was dramatically suppressed (Figures 3(j)–3(o)).

**Figure 4:**
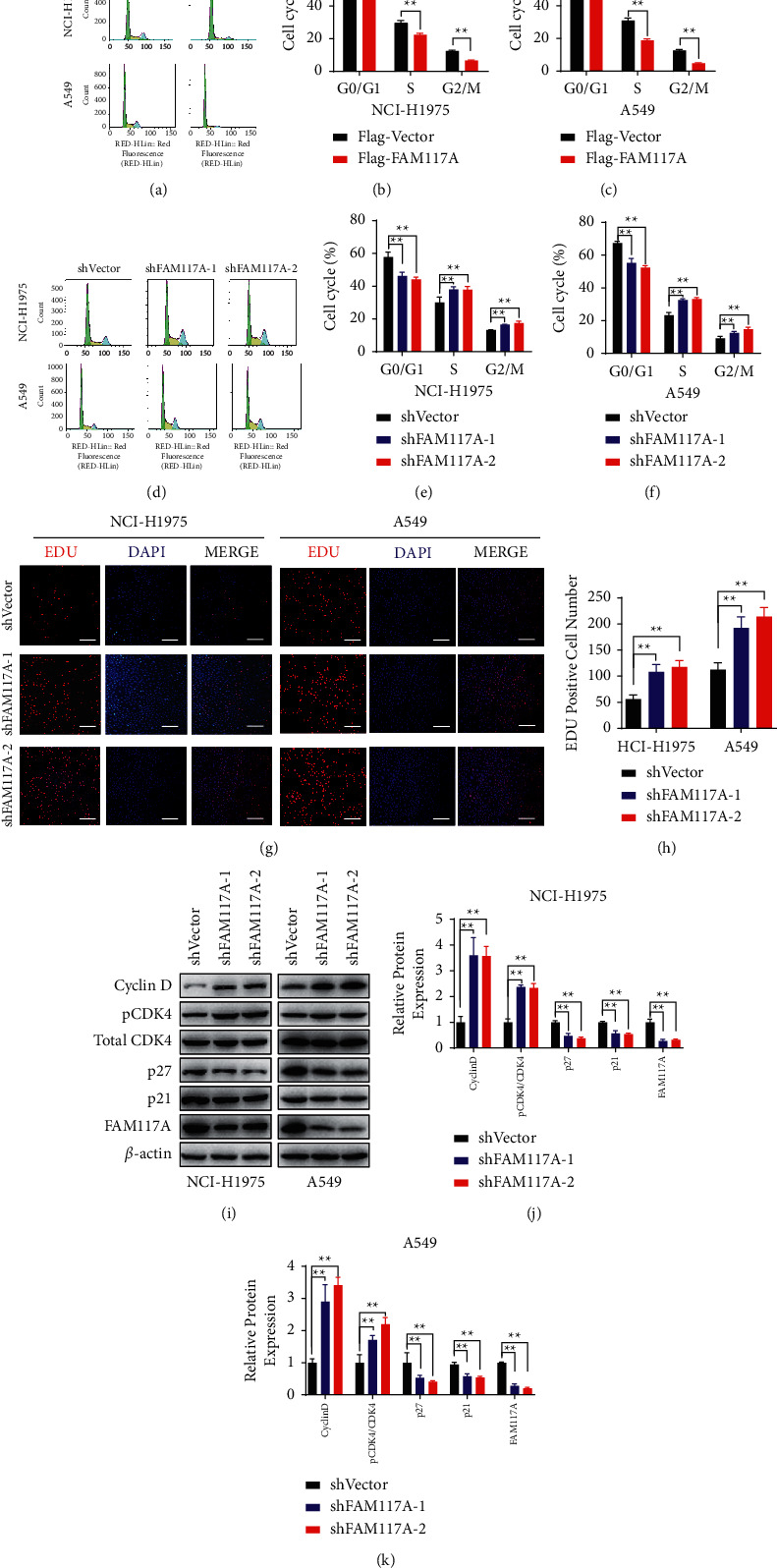
FAM117A regulates the cell cycle progress of lung cancer cells. (a–c) Representative images of the cell cycle profile determined by flow cell cytometry in lung cancer cells transfected with negative control oligo or shRNA oligos targeting the FAM117A transcript. Quantitative analysis results were shown in B (NCI-H1975) and C (A549). (d–f) Representative images of the cell cycle profile determined by flow cell cytometry in lung cancer cells transfected with an empty vector or Flag-FAM117A transcript. Quantitative analysis results were shown in B (NCI-H1975) and C (A549). (g–h) Representative images of EDU labelling lung cancer cells captured by an immunofluorescence microscope. (h, i) Quantitative analysis results of western blot results of FAM117A expression in lung cancer cell lines (NCI-H1975 and A549) upon transfection with Flag-Vector or Flag-FAM117A. Cell cycle-related markers were detected as indicated, and *β*-actin was used as an internal control. (j, k) Quantitative analysis result of the grey scale of the western blot result.

The next set of questions were to identify how FAM117A regulates lung cancer cell growth. To this end, the cell cycle profiles were analyzed by flow cell cytometry in cells with different levels of FAM117A expression. Interestingly, FAM117A overexpression triggered cell cycle arrest at the G0/G1 phase, compared with the control cells (Figures 4(a)–4(c)). On the contrary, FAM117A knockdown leads to accumulated cells in both the S and G2/M phases (Figures 4(d)–4(f)), suggesting that tumor cells were at fast growth state. In addition, the cycle modulation by FAM117A expression was further supported by evidence from the EDU labelling assay, demonstrating decreased EDU signaling in FAM117A overexpressed lung cancer cells and increased in FAM117A silenced cells (Figures 4(g) and 4(h)). Regarding that the cell cycle was precisely regulated by cyclin-dependent kinases (CDKs), we then explore if FAM117A affects the activation of CDK4, a master regulator of G1/S phase. As observed in Figures 4(i)–4(k), phosphorylation of CDK4 at Thr-172 was inversely correlated with the expression level of FAM117A. Besides, the expression of cyclin D was increased upon FAM117A depletion (Figures 4(i)–4(k)). Inversely, the expression of classical cell cycle inhibitors (p21 and p27) was both decreased in lung cancer cells with decreased FAM117A expression (Figures 4(i)–4(k)). In summary, FAM117A might be a negative regulator of lung cancer cell cycle progression.

### 3.4. The Accelerated Cell Cycle Progression Induced by FAM117A Knockdown Could be Rescued by CDK4/6 Inhibitor

**Figure 5:**
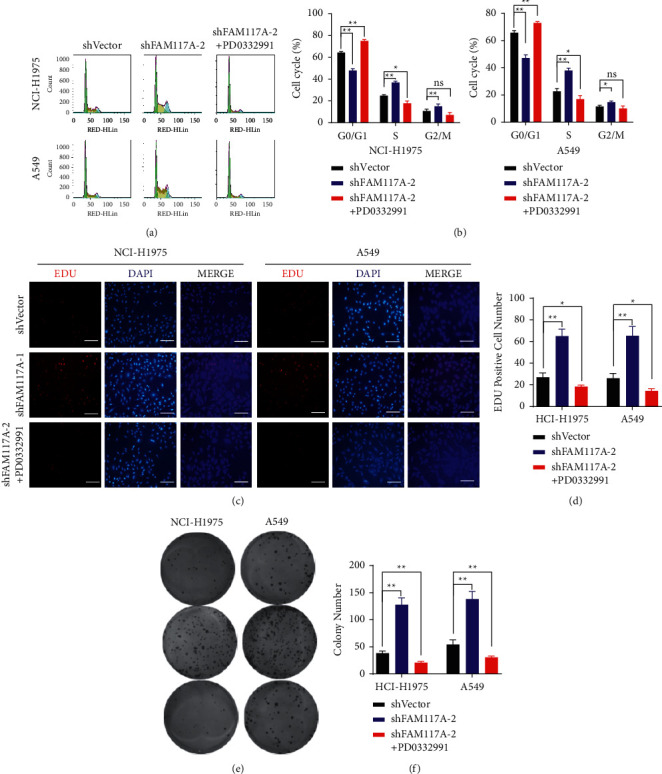
PD0332991 rescued the accelerated cell growth induced by FAM117A knockdown. (a) Representative images of the cell cycle profile determined by flow cell cytometry in lung cancer cells transfected with negative control oligo or shRNA oligos targeting the FAM117A transcript or combined treated with PD0332991. (b) Quantitative analysis results of cell cycle analysis. (c, d) Representative images of EDU labelling lung cancer cells (NCI-H1975 and A549 treated with PD0332991 at 100 nM or DMSO) captured by immunofluorescence microscope. (d) Quantitative analysis results.(e, f) Images of colony formation and quantitative results for lung cancer cells (NCI-H1975 and A549 treated with PD0332991 at 100 nM.

Aberrantly cell cycle progression is a well-established hallmark of cancer, and targeted therapies have been developed to restore cell cycle control for cancer treatment, such as breast cancer [21]. Considering that the cell cycle from G1 to S phase was dramatically promoted in lung cancer cells with decreased FAM117A expression, we next checked whether this can be restrained by CDK4/6 inhibition. To this end, lung cancer cells were treated with PD0332991, which is a well-known small molecular inhibitor of both CDK4 and CDK6 kinases. Interestingly, the accelerated cell cycle profile could be fully reversed by PD0332991 as demonstrated by both flow cell cytometry (Figures 5(a) and 5(b)) as well as EDU labelling (Figures 5(c) and 5(d)). Accordingly, lung cancer cell growth induced by FAM117A knockdown was also fully suppressed upon treatment with PD0332991 (Figures 5(e) and 5(f)). Collectively, decreased FAM117A expression might be a new biomarker for determining the sensitivity of lung cancer patients to PD0332991.

## 4. Discussion

Lung cancer is one of the most malignant form of neoplasm threatening human health [1]. Although targeted therapy has significantly improved the overall survival of lung cancer patients during the past decade, drug resistance is inevitable for most cases [22]. Therefore, new biomarkers for precision medicine must be warranted. Loss of cell cycle control is a classical characteristic of cancer, mostly due to the inability of cell cycle suppressing machines, leading to uncontrolled tumor cell growth [23]. Identification of aberrant regulators for cell cycle progress helps in the development of new therapies for cancer treatment.

In the current study, we identified FAM117A as a new regulator for cell cycle control through bioinformatics analysis of transcriptome data of lung cancer patients. FAM117A was originally identified as a C/EBP-induced protein, and its physiological association with the DNA damage response complex has been reported recently; however, no biological function or molecular mechanism has been reported [20]. In our present study, silencing the expression of FAM117A in lung cancer cells leads to decreased expression of p21 and p27 but increased expression of cyclin D, leading to accelerated cell cycle progression and cancer development. In addition, reexpression of FAM117A in cancer cells might restore the cell cycle checkpoint control, not only in lung cancer cells but also in other cancers.

Clinical benefits have been achieved by targeting cell cycle dependent kinases among different types of cancers [24]. Several antagonists with excellent activity against CDK4 and CDK6 have been approved for treatment of breast cancer, which restore cell cycle control at the G1/S transition and significantly extend patients' lives [12, 25–28]. Furthermore, the clinical benefits of these inhibitors for lung cancer patients are currently under evaluation, and promising results have been observed in certain subtypes of lung cancer patients [29–31]. In the current study, we found that decreased FAM117A expression in lung cancer cells warrants cell cycle progression by releasing the G1/S checkpoint, which could be fully rescued by CDK4/6 inhibitors and provides a molecular basis for treatment of lung cancer patients with CDK inhibitors or other cell cycle modulators. Collectively, our findings provide new evidence for FAM117A as an unfavorable prognostic marker for lung cancer patient survival and might be a new biomarker to predict the sensitivity to CDK4/6 inhibitors.

## Data Availability

All the data generated in this article are available from the corresponding author on reasonable request.

## Abbreviations

TCGA: The Cancer Genome Atlas
FAM117A: Family with sequence similarity 117 member A
LUAD: Lung adenocarcinoma
KEGG: Kyoto Encyclopedia of Genes and Genomes
GO: Gene oncology
GSEA: Gene set enrichment analysis.

## Supplementary Materials

Supplementary Materials Supplemental Figure 1: expression of FAM117A among different types of cancers. Supplemental Table 1: list of whole transcriptome screens of survival-related genes. Supplemental Table 2: correlation between FAM117A expression and clinical features of lung cancer patients.
