# Supplementary material for: FAM117A Is a New Prognostic Marker of Lung Adenocarcinoma and Predicts Sensitivity to PD0332991
Source: Evid Based Complement Alternat Med. 2022 Mar 3;2022:3945446. doi: 10.1155/2022/3945446 (PMC8913056; doi:10.1155/2022/3945446)
Supplement: Supplementary Materials — Supplemental Figure 1: expression of FAM117A among different types of cancers. Supplemental Table 1: list of whole transcriptome screens of survival-related genes. Supplemental Table 2: correlation between FAM117A expression and clinical features of lung cancer patients. [file 3945446.f1.docx]

**Supplemental Table 1. List of whole transcriptome screen of survival related genes.**

| **TCGA ID** | **TCGA Name** | **Updated ID** | **Updated Name** | **Cox coefficient** | **Raw p-value** | **BH-adjusted p-value** | **Median Expression** | **Mean Expression** |
| --- | --- | --- | --- | --- | --- | --- | --- | --- |
| 81558 | FAM117A | 81558 | FAM117A | -0.47 | 2.30E-08 | 0.0004 | 421.4 | 474.7 |
| 27124 | INPP5J | 27124 | INPP5J | -0.44 | 1.20E-07 | 0.0007 | 109.4 | 151.9 |
| 83850 | ESYT3 | 83850 | ESYT3 | -0.40 | 1.30E-06 | 0.0016 | 185.5 | 283.1 |
| 1586 | CYP17A1 | 1586 | CYP17A1 | -0.40 | 4.10E-07 | 0.0009 | 1.4 | 8.4 |
| 55244 | SLC47A1 | 55244 | SLC47A1 | -0.39 | 1.00E-06 | 0.0015 | 159.5 | 316.2 |
| 29116 | MYLIP | 29116 | MYLIP | -0.39 | 2.40E-07 | 0.0008 | 749.4 | 830.2 |
| 284439 | SLC25A42 | 284439 | SLC25A42 | -0.38 | 1.30E-06 | 0.0016 | 250.2 | 270.9 |
| 2788 | GNG7 | 2788 | GNG7 | -0.38 | 1.10E-06 | 0.0015 | 147.0 | 169.1 |
| 27232 | GNMT | 27232 | GNMT | -0.38 | 2.10E-06 | 0.0023 | 6.3 | 13.4 |
| 58473 | PLEKHB1 | 58473 | PLEKHB1 | -0.38 | 6.40E-07 | 0.0011 | 645.6 | 857.2 |

**Supplemental Table 2. Correlation between FAM117A expression and clinical feature of lung cancer patients.**

| **Query** | **Statistic** | **P-value** | **FDR (BH)** |
| --- | --- | --- | --- |
| overall_survival (Cox Regression Test) | -6.27E-01 | 1.81E-09 | 2.17E-08 |
| pathologic_stage (Kruskal-Wallis Test) | 3.12E+01 | 7.80E-07 | 4.68E-06 |
| pathology_T_stage (Kruskal-Wallis Test) | 2.95E+01 | 1.77E-06 | 7.07E-06 |
| pathology_N_stage (Kruskal-Wallis Test) | 2.26E+01 | 4.87E-05 | 1.46E-04 |
| radiation_therapy (Wilcox Test) | -1.39E-02 | 2.78E-02 | 6.68E-02 |
| histological_type (Kruskal-Wallis Test) | 1.92E+01 | 5.79E-02 | 1.08E-01 |
| years_to_birth (Spearman Correlation) | 8.59E-02 | 6.28E-02 | 1.08E-01 |
| pathology_M_stage (Wilcox Test) | -1.37E-02 | 1.94E-01 | 2.91E-01 |
| Tumor_purity (Spearman Correlation) | -1.74E-02 | 7.01E-01 | 8.58E-01 |
| residual_tumor (Kruskal-Wallis Test) | 4.23E-01 | 8.09E-01 | 8.58E-01 |
| race (Kruskal-Wallis Test) | 8.08E-01 | 8.48E-01 | 8.58E-01 |
| ethnicity (Wilcox Test) | 1.89E-03 | 8.58E-01 | 8.58E-01 |

**Supplemental Figure 1. Expression of FAM117A among different types of cancers.**

**
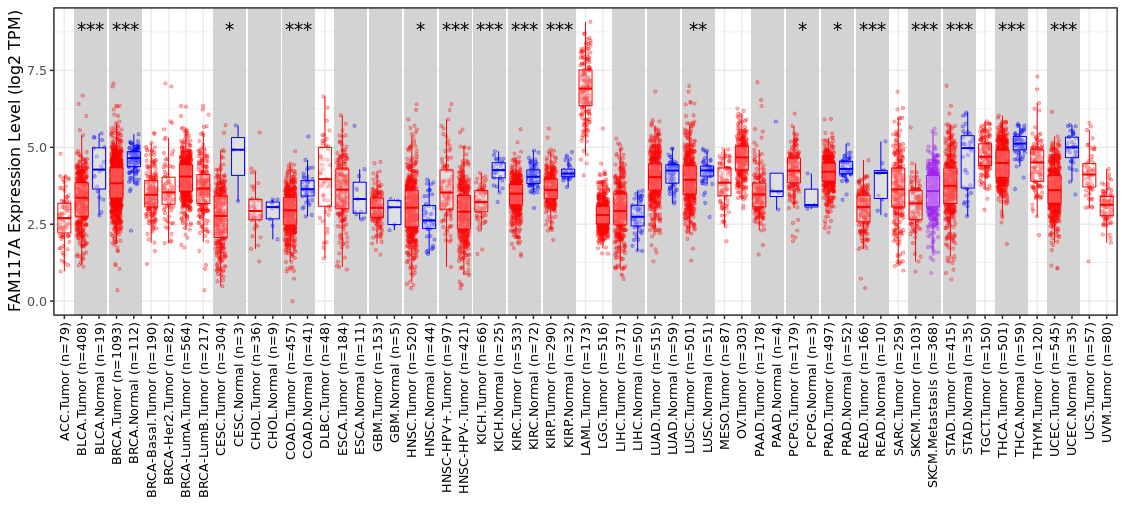
**
